# Gas Geyser-Related Carbon Monoxide Poisoning Presenting as Stroke Mimic and Syncope in Closed-Space Exposure: A Report of Two Cases

**DOI:** 10.7759/cureus.108695

**Published:** 2026-05-12

**Authors:** Ramireddy Gari Hareeth Reddy, Vismaya Mrganayani, Chirag Somasundar, Sai Surya Teja B, Faisal N

**Affiliations:** 1 Emergency Medicine, JSS Academy of Higher Education and Research, Mysuru, IND; 2 General Medicine, Pinnamaneni Siddhartha Medical College, Vijayawada, IND

**Keywords:** carbon monoxide poisoning, carboxyhemoglobin, environmental exposure, gas geyser syndrome, neurological emergency, pulse oximetry limitation, stroke mimic, syncope

## Abstract

Carbon monoxide poisoning is a potentially fatal yet frequently overlooked condition owing to its vague clinical manifestations and deceptively normal pulse oximetry readings. Exposure related to gas geysers represents an under-recognized source of carbon monoxide toxicity in the Indian subcontinent, particularly in confined and poorly ventilated bathroom environments.

We describe two patients with acute carbon monoxide poisoning presenting with different neurological emergencies. The first case involved a 58-year-old man with a history of prior stroke who presented with altered mental status and generalized weakness, initially raising suspicion of an acute cerebrovascular event. The second case involved a 45-year-old woman who developed syncope preceded by tremors and headache while bathing. In both instances, pulse oximetry readings were normal (98%) despite significant underlying hypoxia. Arterial blood gas analysis with CO-oximetry confirmed elevated carboxyhemoglobin levels (33% and 24.1%, respectively).

Administration of high-flow normobaric oxygen led to rapid symptomatic improvement and full recovery in both patients, without the need for hyperbaric oxygen therapy. These cases highlight important diagnostic challenges, particularly the unreliability of pulse oximetry and the critical role of environmental history in identifying carbon monoxide exposure.

Gas geyser-associated carbon monoxide poisoning should be considered in patients presenting with unexplained neurological symptoms or syncope in enclosed bathroom settings. Prompt recognition and treatment are essential, as this represents a preventable cause of significant morbidity and mortality.

## Introduction

Carbon monoxide is a colorless, odorless, and non-irritant gas produced by the incomplete combustion of carbon-containing fuels such as liquefied petroleum gas, wood, and coal [1]. It remains one of the leading causes of accidental poisoning worldwide, contributing significantly to morbidity and mortality across both developed and developing regions [1,2]. The clinical presentation of carbon monoxide poisoning is often non-specific and highly variable, ranging from mild symptoms such as headache, dizziness, and nausea to severe manifestations including altered sensorium, seizures, cardiovascular instability, and even death [3]. This broad clinical spectrum frequently leads to misdiagnosis or delayed recognition, particularly in emergency settings where more common neurological or cardiac conditions are initially suspected.

In high-income countries, carbon monoxide exposure is most commonly associated with house fires, faulty heating systems, or occupational hazards [2]. In contrast, in low- and middle-income settings, particularly in the Indian subcontinent, domestic exposure from gas-powered water heaters represents an important yet under-recognized source of poisoning. These appliances are frequently installed in bathrooms with inadequate ventilation, and their use increases during colder months.

During winter, bathrooms are often intentionally sealed to prevent heat loss, resulting in significantly reduced air exchange. In such enclosed environments, oxygen levels progressively decline during combustion. Under these oxygen-deficient conditions, fuel combustion becomes incomplete, leading to the increased production of carbon monoxide rather than carbon dioxide [1]. The continuous operation of gas-powered water heaters in this setting promotes the rapid accumulation of carbon monoxide within the confined space. As carbon monoxide is non-irritant and undetectable by human senses, exposure often occurs unnoticed until clinical symptoms develop.

This reproducible sequence, enclosed space, reduced ventilation, oxygen depletion, incomplete combustion, and subsequent accumulation of carbon monoxide, creates a high-risk environment for acute toxicity within minutes. This phenomenon, commonly referred to as the so-called gas geyser syndrome, represents a preventable yet clinically significant cause of carbon monoxide poisoning, particularly in resource-limited settings where awareness and safety regulations may be inadequate.

## Case presentation

Case 1

A 58-year-old man was brought to the emergency department following a sudden collapse while bathing in a small, enclosed bathroom fitted with a gas-powered water heater. The bathroom ventilation openings had been sealed due to cold weather.

He had a past medical history of left middle cerebral artery infarction five months earlier, with residual right-sided hemiparesis and mild expressive aphasia. Given this history, a recurrent cerebrovascular event was initially suspected.

There was no clear history of prodromal symptoms such as headache, dizziness, nausea, vomiting, visual disturbance, or chest discomfort prior to collapse.

On examination, the patient was awake but disoriented, with a Glasgow Coma Scale score of E3V3M4 (10/15). Oxygen saturation was 98% on room air, blood pressure was 130/80 mmHg, heart rate was 88 beats/minute, respiratory rate was 18 breaths/minute, and the patient was afebrile. Neurological examination revealed no new focal deficits.

Computed tomography (CT) perfusion imaging demonstrated no evidence of acute ischemia or infarction. Environmental history subsequently raised suspicion of carbon monoxide exposure.

Arterial blood gas analysis with CO-oximetry revealed a carboxyhemoglobin (COHb) level of 33%. Arterial pH was 7.41, and lactate was 1.15 mmol/L.

The patient was immediately started on 100% oxygen via a non-rebreather mask. Repeat testing approximately six hours later demonstrated a reduction of COHb to 1.7%, with progressive return to baseline neurological status.

Although transient neurological manifestations and elevated COHb levels represented relative indications for hyperbaric oxygen therapy, the patient demonstrated rapid clinical and biochemical improvement with normobaric oxygen therapy, without persistent neurological impairment or hemodynamic instability. Immediate access to hyperbaric oxygen facilities was also limited; therefore, conservative management with high-flow oxygen therapy alone was continued.

At the initial follow-up approximately three weeks post-discharge, the patient demonstrated no evidence of delayed neuropsychological sequelae. Repeat evaluation at five months confirmed sustained neurological stability without cognitive impairment, behavioral changes, gait disturbance, or new focal neurological deficits [3,4]. A chronological summary of major clinical events in Case 1 is provided in Table 1.

**Table 1:** Timeline of clinical events: Case 1 Chronological summary of exposure, symptom onset, diagnostic evaluation, treatment initiation, clinical recovery, and follow-up in Case 1 involving gas geyser-related carbon monoxide poisoning presenting as a stroke mimic.

Clinical event	Approximate time
Exposure to a gas geyser in an enclosed bathroom	~06:00
Symptom onset/collapse	~06:30
Emergency department arrival	~07:15
Initial neurological evaluation and computed tomography perfusion imaging	~07:30-08:15
Arterial blood gas with CO-oximetry performed	~08:30
Carboxyhemoglobin level reported (33%)	~08:45
High-flow oxygen therapy initiated	~09:00
Repeat carboxyhemoglobin measurement (1.7%)	~15:00
Neurological recovery to baseline	Same day
Discharge after observation	Next day
Initial follow-up assessment	3 weeks post-discharge
Extended follow-up assessment	5 months post-exposure
Delayed neuropsychological sequelae	None reported

Case 2

A 45-year-old previously healthy woman presented with syncope following exposure to a gas-powered water heater in a sealed bathroom environment.

Prior to collapse, she experienced headache, tremors, and generalized weakness. The tremors were bilateral, generalized, and non-rhythmic, lasting several minutes before resolving spontaneously without anticonvulsant therapy. There was no tongue bite, urinary incontinence, focal motor activity, or postictal confusion, making a primary seizure disorder less likely.

On arrival, she was alert and hemodynamically stable, with a Glasgow Coma Scale score of 15/15. Oxygen saturation was 98% on room air, blood pressure was 120/78 mmHg, heart rate was 84 beats/minute, respiratory rate was 16 breaths/minute, and the patient was afebrile.

Electrocardiography demonstrated subtle ST-T abnormalities in the anterior precordial leads (V2-V6), including mild ST-segment flattening and shallow T-wave inversions (Figure 1), consistent with transient myocardial hypoxia. Echocardiography showed preserved ventricular function without regional wall motion abnormalities.

**Figure 1:**
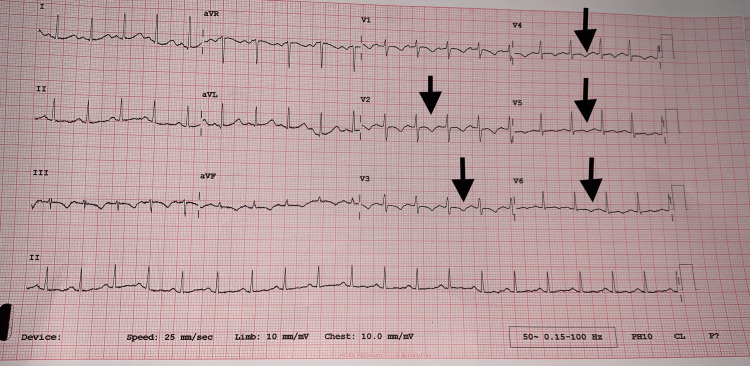
Electrocardiographic changes in carbon monoxide poisoning 12-lead electrocardiogram demonstrating subtle ST-T abnormalities in leads V2-V6 (highlighted by straight digital arrows), including mild ST-segment flattening and shallow T-wave inversions, suggestive of transient myocardial ischemia or non-specific repolarization abnormalities associated with carbon monoxide exposure.

Arterial blood gas analysis with CO-oximetry revealed a COHb level of 24.1%. Arterial pH was 7.39, and lactate was 0.9 mmol/L. Troponin testing was not available at the time of presentation.

The patient was treated with high-flow oxygen therapy, resulting in rapid clinical improvement. Repeat COHb levels decreased to 1.4%, and the electrocardiographic abnormalities resolved completely.

Although transient loss of consciousness and elevated COHb levels represented relative indications for hyperbaric oxygen therapy, the patient demonstrated rapid symptomatic improvement without persistent neurological impairment, cardiovascular instability, or metabolic acidosis. In addition, immediate access to hyperbaric oxygen therapy was limited; therefore, management with normobaric oxygen therapy alone was continued.

At the three-week follow-up, the patient remained asymptomatic without evidence of delayed neuropsychological sequelae. Repeat assessment at five months demonstrated continued clinical stability without recurrent neurological or cognitive symptoms. A chronological summary of major clinical events in Case 2 is provided in Table 2.

**Table 2:** Timeline of clinical events: Case 2 Chronological summary of exposure, symptom progression, diagnostic evaluation, treatment course, recovery, and follow-up in Case 2 involving gas geyser-related carbon monoxide poisoning presenting with syncope and transient cardiac manifestations.

Clinical event	Approximate time
Exposure to a gas geyser in an enclosed bathroom	~07:00
Onset of headache, tremors, and weakness	~07:15
Transient loss of consciousness	~07:20
Recovery of consciousness	~07:40
Emergency department arrival	~08:15
Initial clinical evaluation and electrocardiogram	~08:30
Arterial blood gas with CO-oximetry performed	~08:45
Carboxyhemoglobin level reported (24.1%)	Shortly after testing
High-flow oxygen therapy initiated	Immediately after diagnosis
Repeat carboxyhemoglobin measurement (1.4%)	Approximately 6 hours later
Resolution of electrocardiogram abnormalities	Same day
Clinical recovery	Same day
Discharge after observation	Next day
Initial follow-up assessment	3 weeks post-discharge
Extended follow-up assessment	5 months post-exposure
Delayed neuropsychological sequelae	None reported

A comparative summary of the clinical characteristics, investigations, treatment, and outcomes of both cases is presented in Table 3.

**Table 3:** Comparative summary of clinical characteristics between both cases Side-by-side comparison of demographic characteristics, presenting symptoms, clinical findings, laboratory parameters, treatment, and outcomes in both patients with gas geyser-related carbon monoxide poisoning. GCS: Glasgow Coma Scale; ECG: electrocardiogram; CT: computed tomography; COHb: carboxyhemoglobin; NRBM: non-rebreather mask; DNS: delayed neurological sequelae

Variable	Case 1	Case 2
Age/sex	58/M	45/F
Exposure setting	Enclosed bathroom with sealed ventilation	Enclosed bathroom with sealed ventilation
Exposure source	Gas-powered water heater	Gas-powered water heater
Presentation	Collapse, altered sensorium, stroke mimic	Syncope, tremors
Prodromal symptoms	No prodromal symptoms clearly reported	Headache, tremors, weakness
GCS	E3V3M4 (10/15)	15/15
Blood pressure	130/80 mmHg	120/78 mmHg
Heart rate	88 beats/minute	84 beats/minute
Respiratory rate	18 breaths/minute	16 breaths/minute
Temperature	Afebrile	Afebrile
Oxygen saturation	98% on room air	98% on room air
ECG findings	No acute abnormality	ST-T abnormalities with T-wave inversions (V2-V6)
CT imaging	No acute infarction	Not required
COHb level	33%	24.1%
Arterial pH	7.41	7.39
Lactate	1.15 mmol/L	0.9 mmol/L
Troponin	Not available	Not available
Treatment	100% oxygen via NRBM	High-flow oxygen therapy
Repeat COHb	1.7%	1.4%
Hyperbaric oxygen therapy	Not administered	Not administered
Outcome	Full neurological recovery	Full recovery
DNS at follow-up	None at 5 months	None at 5 months

The patient had been bathing in a small, enclosed bathroom with a gas-powered water heater and inadequate ventilation (Figure 2). The bathroom environment was characterized by limited air exchange and the absence of external exhaust, creating conditions conducive to oxygen depletion and incomplete combustion. This enclosed setup represents a typical high-risk environment for carbon monoxide accumulation.

**Figure 2:**
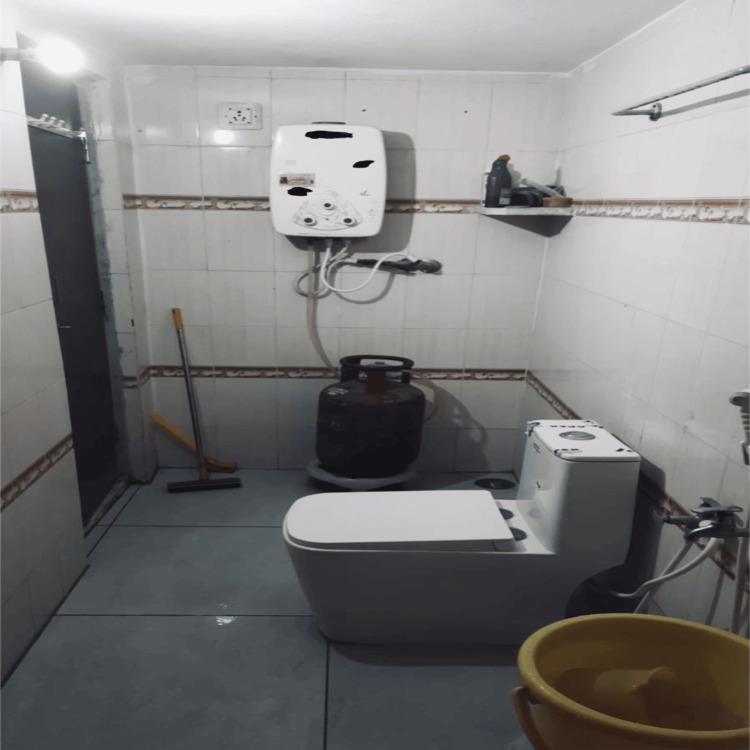
Enclosed bathroom environment with a gas-powered water heater Photograph demonstrating a poorly ventilated bathroom setup with a gas-powered water heater installed in an enclosed space, illustrating a typical high-risk environment for carbon monoxide accumulation.

## Discussion

Carbon monoxide toxicity begins at the level of environmental exposure, most commonly in enclosed spaces where incomplete combustion of carbon-containing fuels leads to the rapid accumulation of carbon monoxide [1,2]. In the setting of gas-powered water heaters, combustion ideally produces carbon dioxide and water; however, under oxygen-deficient conditions, such as poorly ventilated or sealed bathrooms, combustion becomes incomplete, resulting in the generation of carbon monoxide. This closed-space, low-oxygen environment promotes the continuous production and accumulation of carbon monoxide, leading to toxic exposure within minutes. This sequence forms the basis of the so-called gas geyser syndrome and represents the central pathogenic mechanism in the present cases.

Following inhalation, carbon monoxide rapidly diffuses across the alveolar-capillary membrane and binds to hemoglobin with an affinity approximately 200-250 times greater than oxygen, forming COHb [1]. This markedly reduces oxygen-carrying capacity and shifts the oxyhemoglobin dissociation curve to the left, impairing oxygen release at the tissue level. Consequently, significant tissue hypoxia may occur despite apparently normal oxygen saturation readings, a phenomenon observed in both cases [5].

At the cellular level, carbon monoxide exerts direct toxic effects by inhibiting cytochrome c oxidase within the mitochondrial electron transport chain, thereby disrupting oxidative phosphorylation and reducing adenosine triphosphate production [3]. This leads to cellular hypoxia, oxidative stress, and lipid peroxidation, resulting in injury to metabolically active tissues such as the brain and myocardium [3,4]. These mechanisms explain both the acute neurological manifestations and the risk of delayed neurocognitive sequelae [3,4].

The diagnostic process is frequently complicated by the non-specific nature of symptoms and the limitations of standard monitoring tools. Pulse oximetry is unreliable in this context because standard devices cannot differentiate COHb from oxyhemoglobin and may produce falsely reassuring oxygen saturation readings [5]. 

Once recognized, prompt management is critical. Administration of high-flow oxygen accelerates the dissociation of carbon monoxide from hemoglobin and improves tissue oxygenation [1]. In selected cases with severe toxicity, hyperbaric oxygen therapy may be considered, particularly in patients with neurological impairment or elevated COHb levels [6,7]. Further cellular injury mechanisms include mitochondrial dysfunction and oxidative stress, as described in experimental and clinical studies [8].

Although both patients in the present report demonstrated relative indications for hyperbaric oxygen therapy, including transient neurological manifestations, loss of consciousness, and elevated COHb levels, neither exhibited persistent neurological impairment, hemodynamic instability, severe metabolic acidosis, or evidence of ongoing end-organ dysfunction following the initiation of treatment. Both patients demonstrated rapid clinical and biochemical improvement with immediate administration of high-flow normobaric oxygen therapy, including substantial reduction in COHb levels within hours of presentation. In addition, immediate access to hyperbaric oxygen facilities was limited at our institution. Based on these factors, conservative management with normobaric oxygen therapy alone was considered appropriate in both cases.

As hypoxia progresses, clinical manifestations become evident. Neurological symptoms such as altered sensorium, syncope, and seizures are common, while cardiac involvement may manifest as arrhythmias or transient electrocardiographic abnormalities [9]. In the present report, Case 1 presented as a stroke mimic, whereas Case 2 demonstrated subtle ST-T changes, highlighting the diverse and often misleading clinical presentations of carbon monoxide poisoning.

From a diagnostic perspective, carbon monoxide poisoning should be considered in patients presenting with unexplained neurological or cardiovascular symptoms occurring in enclosed environments, particularly when multiple individuals are exposed to a common combustion source. Because pulse oximetry may remain falsely reassuring, confirmation of diagnosis relies on CO-oximetry for the accurate measurement of COHb levels [10].

Both patients underwent follow-up assessment at approximately three weeks post-discharge and again at five months, with neither demonstrating evidence of delayed neuropsychological sequelae. Nevertheless, delayed complications may manifest days to weeks following exposure and remain an important consideration requiring patient counselling and follow-up [3,4].

Certain clinical parameters, including serial cardiac biomarkers and formal neurocognitive assessment scales, were not uniformly available in both cases. Troponin testing was unavailable in Case 2 at the time of presentation. These limitations reflect the retrospective nature of clinical data collection in emergency settings.

## Conclusions

Gas geyser-related carbon monoxide poisoning represents a preventable yet potentially life-threatening cause of neurological and cardiovascular morbidity, particularly in poorly ventilated enclosed environments. The present cases demonstrate that carbon monoxide poisoning may present atypically as a stroke mimic, altered sensorium, syncope, or transient electrocardiographic abnormalities despite apparently normal pulse oximetry readings.

These cases further highlight the diagnostic importance of careful environmental exposure history and early consideration of carbon monoxide toxicity in patients presenting with unexplained neurological or cardiovascular symptoms in enclosed spaces. Prompt recognition and initiation of high-flow oxygen therapy resulted in rapid clinical and biochemical recovery in both patients, with no evidence of delayed neuropsychological sequelae at extended follow-up.

Greater awareness regarding domestic gas geyser-related carbon monoxide exposure, appropriate ventilation practices, and early use of CO-oximetry may facilitate earlier diagnosis and help prevent avoidable morbidity and mortality.
